# The influences of background on beginning medical students’ perceptions of rural medical practice

**DOI:** 10.1186/s12909-015-0339-9

**Published:** 2015-03-25

**Authors:** Robin A Ray, Louise Young, Daniel B Lindsay

**Affiliations:** 1 College of Medicine and Dentistry, James Cook University, Townsville, 4811 Australia; 2 Department of Psychology, College of Health Care Sciences, James Cook University, Townsville, 4811 Australia

**Keywords:** Rural medical practice, Rural background, Urban background, Perceptions, Medical students, Communication, Selection

## Abstract

**Background:**

Access to health care is an ongoing problem for underserved populations in rural and remote regions of Australia and North America. Despite medical schools educating more medical doctors, this maldistribution continues. While students entering medical programs with a rural focus purport to have an interest in rural medicine, their understanding of possible future rural practice is unclear. This study explored the differences in perception of rural practice between beginning medical students from rural and urban backgrounds to gain an indication of the usefulness of our selection process to meet the rural workforce mandate.

**Methods:**

Beginning medical students completed a writing exercise about the life and work of a rural medical doctor as a test of their academic writing skills. After completing the task and receiving feedback, students were invited to submit their work for analysis. Template analysis using themes from a study of rural medical registrars was used to analyse 103 scripts.

**Results:**

Students demonstrated foundational insight into some of the realities of rural life and practice. However, differences were noted in perspectives between rural background students and urban background students. Rural background students used everyday language to describe the practicalities of rural life, medical practice and the implications for families and communities. Urban background students generally used complex language and more negative descriptors.

**Conclusions:**

Beginning medical students from urban and rural backgrounds differ in their perceptions and expression of rural practice. These outcomes are important for medical schools that use interviews in their selection process. Rural background applicants’ suitability may be overlooked because of the interviewer’s expectations of language, while urban background applicants may score higher related to complex language and use of key phrases. Interviewer training should address this likely bias thereby increasing the potential to recruit rural background students.

## Background

James Cook University (JCU) in North Queensland, Australia, established an undergraduate medical program in 2000 with a mission to select and educate medical graduates prepared to work as medical doctors in rural and remote locations [1]. While a range of students from urban and rural backgrounds are attracted to the JCU course, the selection process purposively recruits students from rural areas, considers rurally adjusted academic achievement and interviews applicants to identify attributes relevant to rural medical practice. The medical curriculum is underpinned by a culture of altruism, infused with rural and Indigenous content and delivered with a generalist focus.

Rural background is known to be a significant contributor to graduates working in rural areas [2,3]. Additionally, students from urban backgrounds may be influenced by the rural experiences in a medical course [4,5]. However, little is known the about the differences between urban background and rural background students’ perceptions of rural medical practice and how these are expressed in the early stages of their medical training.

In Australia in 2012, 15.0 million people (66% of the population) resided in major cities and 7.7 million (34%) resided in the rest of Australia [6]. People in rural areas have access to less than half of the medical practitioners per capita when compared with medical practitioners per capita in metropolitan areas [7]. Similar statistics are found in North America where 19.2 percent of the population, but only 11.4 percent of the nation’s medical doctors, live in rural areas [8]. This maldistribution of medical doctors results in large underserved populations highlighting the need for more medical students to train for careers in rural locations [9].

Over the past 2 decades, the Australian Government has implemented schemes to increase the rural health workforce [10] and Australian medical schools are educating increased numbers of medical students with intakes more than doubling from 1335 in 2006 to a projected 3108 in 2014 [11]. Yet, while there are more qualified medical doctors, there is limited evidence that more medical doctors are working with underserved populations, especially in rural areas where surveys indicated that medical doctors numbers remained stable between 2008 and 2012 [12]. A recent Rural Health Workforce report based on self reporting claimed an 8.1 percentage increase in the number of rural and remote general practitioners. However, as self reporting patterns fluctuate and the rural classification used in the reporting system changed since the last reporting period, this figure needs to be treated with caution [13].

Previous studies have demonstrated that decision making about rural and remote practice is complex, predicated on many factors including rural experience, family issues, spouse with rural background, practice expectations, career pathway opportunities and social factors [3,14,15]. Some evidence suggests that clinical attachments during a medical course also contribute to rural practice intentions. However, evidence from North America and Australia is divided as to whether these attachments reinforce preliminary interests or engender new motivation for rural practice [16,17]. Given that multiple factors influence rural practice, assessing rural practice potential during the admission process remains a topic of conjecture as intent to and the actuality of rural practice are subject to many influences that are not measurable at the time of medical school admission. Selection based on academic achievement and non-cognitive variables such as communication skills and personality traits like empathy and openness is used widely by medical schools [18,19] to select applicants most suited to study and practice modern medicine. However, as most medical students are from higher socio-economic backgrounds many of whom have been educated in non-government schools [20,21], over emphasis on communication may preclude applicants from lower socio-economic backgrounds including rural applicants.

Selection processes at JCU include an interview where applicants have the opportunity demonstrate their suitability for our medical course. Given that JCU has a focus on graduating healthcare professionals to work in rural and remote areas, this study explored the differences in perception of rural practice between first year medical students from rural and urban backgrounds to gain an indication of the usefulness of our selection process to fulfil JCU’s rural workforce mandate.

## Methods

### Recruitment and data collection

During week two of the first year of JCU’s six year undergraduate medical program, students are required to complete an impromptu, defined topic, academic writing exercise. The purpose of this task is to assess students’ academic writing skills to identify those who require remediation. This commonly occurring process was recognised as an ideal opportunity to collect data from these students in an unobtrusive manner.

After consultation with the first year co-ordinator, the beginning students were asked to write about the life and work of a rural medical doctor. Writing about this topic after students had been accepted into medicine avoided the interview response bias common to selection applications [22]. Data were collected during the second week of Bachelor of Medicine Bachelor of Surgery course in 2012 and 2013. Upon completion of the academic writing task, the project was explained to the students, they were given an information sheet and an opportunity to ask questions. After the writing tasks had been assessed, students were reminded about the study, then invited to retain their feedback page and submit their work for analysis.

Ethical clearance for the project was granted by JCU Human Research Ethics Committee. Consent was presumed when students voluntarily submitted their writing exercises through a secure assignment box in the foyer of the Student Education and Placement Unit.

### Data analysis

**Figure 1:**
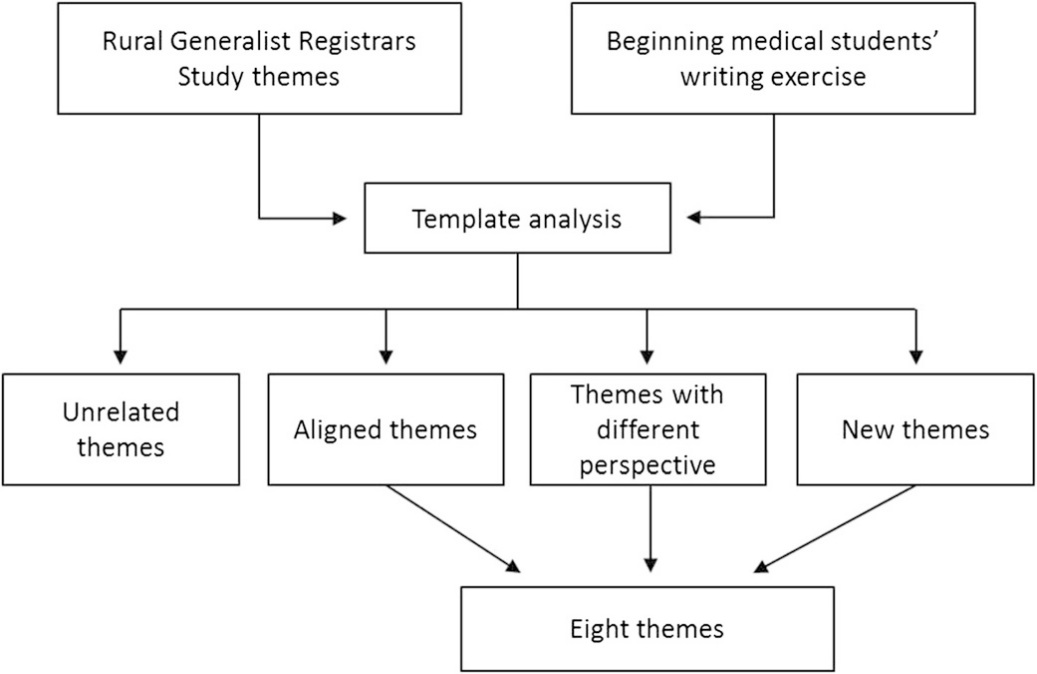
**Template analysis flow chart.** This chart demonstrates the analytical process used in thematic analysis.

Template analysis [23] enabled us to efficiently analyse the large amount of textual data from 103 writing exercises managed in NVivo qualitative software. Two authors (RR and DL) independently applied the processes of combining top down (known themes) and bottom up (identification of new codes), to test the relevance of existing themes and identify new ways of constructing and describing themes (see Figure 1 Template analysis flow chart). We began with a coding frame (template) generated by a previous study of the perceptions of rural generalist registrars [24,25]. Beginning analysis with an a priori template expedited the process as it provided codes and themes that could be compared and tested against the new data. The coding frame was then revised and adapted as new themes and codes arose from the data and existing themes were redefined by the current study [26]. Codes and themes were then compared and discussion with input from the third author (LY) was used to resolve any discrepancies and refine themes.

During the process of analysis, differences in perception about rural practice were noted and we began to consider whether these differences were a result of students’ location of origin. To test this impression, we accessed demographic data for both cohorts and cross referenced this with the student number recorded on each script. This enabled us to compare themes arising from data generated by students from a rural background with data from students with an urban background. Background was determined by home postcode with rural background being further defined as having completed 5 or more years of education in a rural location as classified by the Australian Standard Geographical Classification – Remoteness Areas (ASGC-RA) system (Table 1). These classifications are determined based on population size and the distance of a location from the nearest urban centre in relation to access to goods and services [27].

**Table 1:** **ASGC-RA classification system categories**

ASGC-RA 1	Major Cities of Australia
ASGC-RA 2	Inner Regional Australia
ASGC-RA 3	Outer Regional Australia
ASGC-RA 4	Remote Australia
ASGC-RA 5	Very Remote Australia

Data were collected from students across the spectrum of location classifications with the majority classified as ASGC-RA 3–5 (Table 2). Data from International students were excluded from the analysis as their ASGC-RA equivalent could not be verified.

**Table 2:** **Respondents and non-respondents by background as classified by ASGC-RA**

	Participant	Non-participant		Participant	Non-participant
ASGC-RA 1	24	111	ASGC-RA 3-5	46	123
ASGC-RA 2	21	54	International	12	45

A significant delay in the return of the assessed writing exercise to students in 2013 contributed to a low response rate as students had lost interest in the academic writing feedback and this study despite several attempts to encourage participation.

## Results

Analysis revealed that data generated by entry level medical students from urban backgrounds expressed more negative codes (31) for perceptions of rural medical practice than their peers from rural backgrounds (25 codes). Conversely, data from rural students resulted in just over double the number of positive codes (55/27) (see Table 3).

**Table 3:** **Numbers of codes by theme and background**

Theme	Urban background		Rural background	
	Positive	Negative	Positive	Negative
Rural lifestyle	2	7	9	3
Pressure of the job and isolation	1	12	2	10
Limited resources	2	8	2	5
Dr/patient relationship	3	1	10	3
Diversity of practice	7	1	15	nil
Making difference in community	10	nil	18	nil

### Rural lifestyle

Previous research suggests that lifestyle factors are can be important barriers to the likelihood of rural practice [3,15]. Students from urban backgrounds wrote about difficulties in making a transition from urban living to rural living citing limitations on social life and privacy, the loss access to technology and limited sporting facilities. One student used the word “sacrifice” adding more poignancy to their perception of the lifestyle loss. The impact on families including schooling for their children and employment for partners was also a concern that was supported by rural background students. Regardless of background, students identified difficulties with the life work balance. However, many rural background students also enthusiastically described the opportunities for social interaction suggesting that rural living enabled the building of sustaining relationships that were important for both medical practice and living rurally (Table 4).

**Table 4:** **Theme 1 rural lifestyle**

Urban background	• *“Rural doctors coming from larger cities will have to sacrifice the lifestyle they are used too, including technological advantages and reliance on co-workers and partners.”*
	• *“The main concern faced by many rural doctors…would be adapting to the lifestyle…due to the smaller population, it would be hard for the doctor to separate work and social life and privacy could become a problem.”*
	• *“In comparison to metropolitan areas, there are often significantly less facilities available…These factors can often lead to greater hardships in dealing with stress and finding enjoyment. Also, social interaction can be difficult due to geographic factors, resulting in isolation and in more severe circumstances, depression.”*
	• *“Rural doctors can also face the challenge of finding schools for their children and work for their partner.”*
Rural background	• *“For many, adapting to a small community poses some challenges and rewards…its more difficult to stay at the right balance of casual and professional, placing further stress on the physician.”*
	• *“There is a wonderful social aspect, which allows the doctor to connect with their community and colleagues in a way that would not be possible for a doctor in a metropolitan area.”*
	• *“Obviously not the same variety of entertainment one will find in a city but by forming relationships with the locals, it is easy to maintain a good social life as well.”*
	• *“The singularly most worthwhile aspect of being a rural doctor is the lifestyle. Despite being on call, the laid back, calm rural atmosphere creates an experience that it is better felt than described.”*

### Pressures of the job and isolation

The demands of rural practice were categorised under the themes of pressure and isolation. Most students regardless of background described isolation and job pressure as having a negative impact on the medical doctor (Table 3 indicates 12 negative codes for urban background students and 10 for rural background students). However, urban background students perceived the problems as originating from the need to work long hours with little access to technology and other medical facilities, as well as being on constant call. While the long hours were acknowledged by rural background students, these students added more emphasis to the lack of a professional network to support decision making, the wider impact on quality of care, and the personal toll on the medical doctor in terms of mental health. Community reaction to mistakes was also highlighted by one rural student (Table 5).

**Table 5:** **Themes 2 and 3 pressures of job and isolation**

Urban background	• *“There’s a whole lot of hard work with little acknowledgement. A rural doctor is understaffed, under resourced and constantly fighting a battle that never seems to end.”*
	• *“A rural doctor may be placed on-call more often than a city doctor, or even permanently. This can make it hard to separate work and life for rural doctors*.”
	• *“You are one of the very few doctors within a certain area and may be required to be extensively ‘on call’.”*
	• *“At times you could feel disconnected from the world. Distance from family and technology often hinders and fogs the decision making.”*
Rural background	• *“Rural GPs may also find, particularly in small communities, that there may be a sole practitioner or have limited colleagues …can lead to a sense of professional isolation whereby rural GPs can’t afford the luxury of a direct second opinion.”*
	• *“Rural doctor may be it driving seven hours to run a clinic or being the only doctor in the town hospital....there may be times when isolation can be overwhelming effecting their mental health.”*
	• *“If the doctor makes a wrong decision regarding the treatment of a patient, he would be defamed quite quickly in the community.”*

While the theme of limited resources also generated negative perceptions of rural practice as was expected (Table 3 records 8 negative codes for urban and 5 negative codes for rural); urban background students overtly described more of the stress and strain created: “*being away from other doctors also would then mean more accountability for the doctor which could cause emotional strain and affect their confidence in treatment*”. However, the pervading negative attitudes were balanced by three students of urban background and four students or rural background who expressed the possibilities for innovation and skill development that occur when resources are limited (Table 6).

**Table 6:** **Theme 4 limited resources**

Urban background	• *“The professional skills of a doctor may also benefit from lack of funding and resources, encouraging creative treatment methods and ideas.”*
	• *“Rural hospital is likely to lack the same resources found in a metropolitan area……this limitation forces a doctor to become more innovative, relying on their knowledge and that of their peers to improve patient outcomes.”*
Rural background	• *“…forced to adapt to the new situation; developing new skills that would not be in the skill set of urban doctors …towns with less sophisticated technology will allow for practitioners to develop new skill set.”*
	• *“Rural doctors tend to treat more patients due to the shortages experienced in remote areas. As a result, rural doctors have become efficient in diagnosing patients with the absence of additional evidence by developing history taking skills and additional research skills.”*

### Medical doctor-patient relationship

Most rural background students wrote something about the medical doctor patient relationship, while only half the urban background students mentioned this relationship as part of rural medical practice. The statements from urban background students were more notionally constructed emphasizing the importance of the medical doctor’s position with the patients and his or her standing in the community. While rural background students acknowledged the potential confidentiality issues that arise in small communities, their statements were nuanced more personally reflecting a closer bond between medical doctors and patients that may be continued over a long period of time (Table 7).

**Table 7:** **Theme 5 doctor/patient relationship**

Urban background	• *“It is important to be able to build solid trusting relations with patients as life and work are more closely linked in small communities as friends may also be patients.”*
	• *“Working in a rural community means privacy concerns are more palpable than if the doctor was working in the city.”*
	• *“It is highly important that the doctor presents him/herself in a trusting, helpful and knowledgeable manner in order to gain the respect and trust of community members.”*
Rural background	• *“It is likely that if a patient walks into his office, there would be no need for introductions to be exchanged. However, a small slip of the tongue could lead to a major confidentiality issue which in turn could lessen a communities faith in the doctor.”*
	• *“Rural doctors have a deeper understanding of what their patients do and why their health is important, as a result they are better equipped to treat them.”*
	• *“Provides opportunity to see patients from the beginning to the end of their treatments, forming bonds with the community members and gaining respect and satisfaction.”*

### Diversity of practice

Although rural background students wrote about the diversity of practice three times more often than urban background students, there was little difference in the content expressed by both groups of students. Small variations occurred in a few papers when some of the rural background students provided more specific detail about types of patients or presenting conditions, and one urban student identified the need for additional reading and research to manage the range of practice (Table 8).

**Table 8:** **Theme 6 diversity of practice**

Urban background	• *“A much greater diversity of cases and the lack of speciality doctors means the doctor would see all parts of the medicine branch.”*
	• *“Doctors who work in these areas deal with a variety of different cases that they may not usually treat. A rural doctor will have to do additional reading and research to extend their knowledge of medicine in order to treat such cases.”*
Rural background	• *“Rural work gives rural doctors a chance to practice medicine across a wide variety of disciplines, rather than just one sub-speciality.”*
	• “*Rural doctors handle the diversity of illness due to exposure to livestock and injury though the handling of farm equipment is far greater*.”
	• *“Indigenous health is a priority area for rural doctor, they need to be trained to not only know about the specific health needs of indigenous people, but also work alongside them to promote positive health outcomes.”*

### Making a difference in the community

Data under the theme of making a difference in the community as generated by rural background students again reflected more personal nuances when students described the medical doctor’s relationship with the community as both professionally and socially rewarding, implying a mutuality of benefits. About one third of the urban students wrote under this theme, several using complex language and key phrases without any supporting detail, indicating more of a theoretical approach to rural medical practice (Table 9).

**Table 9:** **Theme making a difference in the community**

Urban background	• *“Rural doctors have a tough life, but their efforts will be forever a mark on an appreciative, under-privileged community.”*
	• *“Rural practice gives you immense satisfaction of knowing you are assisting underserved populations.”*
	• *“Rural areas hold an untapped resource potential, providing rural doctors with the opportunity to contribute to the body of knowledge on rural, remote, tropical and indigenous issues.”*
Rural background	• *“Doctors are able to form close-knit networks with their communities and are often seen as a mentor, listener and even friend.”*
	• *“Working in a small community and developing strong relationships, they will feel they are making a substantial difference to the well-being of the town.”*
	• *“Building of relationships within the community offers the moments that can make a difference to healthy outcomes for the doctor, their family and the community.”*

## Discussion

Our study suggests that medical students entering the JCU medical course which is focused on rural and remote medical practice, begin the course with a mixture of experientially and notionally informed perceptions about their possible future area of practice. Using these two frames (experiential and notional), two outcomes emerged. First, descriptions of the broad diversity of practice, challenges of isolation, long hours and access to resources as documented in other studies, were well represented in our data [4]. Second, while data from students with rural and urban backgrounds reflected both positive and negative codes, none of the students portrayed rural life as an idyllic existence, nor did they overlook the downsides of rural medicine [28].

Most importantly for the selection of medical students for future rural practice, our study highlighted a difference in the way students from rural backgrounds perceive rural life and medical practice when compared with their counterparts from urban backgrounds. Urban background students expressed more negative statements than rural background students and tended to use more complex, sometimes emotive language, while rural background students provided more personally nuanced everyday language descriptions.

### Perceptions of rural practice

Rural background students reflected their lived experience through recognizing more of the practicalities of rural life and the implications for families and communities. Their writing contained specific detail including possible scenarios such as “driving four hours”, and facing the reality of equipment limitations and “making do”. They also saw opportunities for the medical doctor to be innovative and to manage with fewer resources. On the other hand, the writings of urban background students were expressed in more general terms, using key words and phrases often with little explanation. Educationalists propose that spoken language often assumes implicit knowledge and therefore is less detailed, whereas there is an expectation that written language will be more explicit [29]. Applying this finding to the writing of urban background students suggests that their lack of detail expressed in their writing may arise from relying on less intimate knowledge to form their perception of rural practice. This finding indicates a need to provide well supported, purposively structured opportunities for medical students to study and experience rural environments during their course if we are to graduate medical doctors who are prepared for work in rural areas [5].

Urban background students further distinguished themselves from students of rural background through their use of negatively phrased descriptors and an individualistic focus on the medical doctor. Emotive words such as “inferior” when describing treatments available and “ridiculously long hours” to describe the pressure of the job were used by urban background students to portray the gravity of the situation in rural and remote areas. Their writing also reflected the centrality of the medical doctor rather than the medical doctor in a context as was evident in the writings from rural background students; portraying an individualistic rather than a community world view. While this perception does not discount any altruistic intent on the part of these students, it does point to a need to build community awareness and community participation skills into the medical curriculum. At JCU, these skills are built through close interaction with community volunteer patients from semester one of year one, studies in rural and remote health and 20 weeks of rural attachments spread across years two, four and six. Other research indicates the value of extended attachments to establish rural connectedness [3]. However, extending attachments may be an impost for students who need to work to support themselves during their study [30]. While extended rural attachments may be logistically difficult to implement across a student cohort, extended placements offer opportunities for medical students to be immersed in rural communities and the realities of rural practice. The decline in empathy and idealism among medical students identified in previous studies [31,32] maybe redirected through students’ exposure to the hidden curriculum available in this type of placement, influencing attitudes to social justice and idealism [33]. Thus, opportunities available during extended placements could increase the likelihood of medical graduates working in rural and remote, underserved locations.

### Implications for selection

While prior academic achievement is an essential part of medical selection, incorporating interviews undertaken by a panel of medical, academic and community members has also been shown to have some predictive validity when choosing applicants who will make “good” medical doctors [34]. Given that medical selection interviews generally include a rating based on communication, and that the majority of medical applicants come from higher socio-economic backgrounds, a bias towards articulate applicants who have the knowledge and skills to employ key phrases and complex language could be disadvantaging applicants from rural and remote backgrounds [35]. The issue of language could be all the more important in selection interview panels that include senior medical doctors, as a recent study of selection processes for specialties revealed that more emphasis was placed on the use of English, than on the content of the statement [36]. These factors are particularly important for medical schools like JCU, who seek students who are more representative of the populations they are most likely to serve [37]. Our study revealed differences in style of language and word choice between urban and rural background students. Urban background students’ language reflected notional understandings, while more often, rural background students tended to use everyday language. This was very evident when urban background students used words like “underserved populations” and “under-privileged communities” without any supporting detail, as opposed to phrases such as “practising in areas of need”. A halo effect induced by formal language may elicit a more positive general impression, masking a lack of detail in applicant responses to specific questions [38,39]. Additionally, when rural applicants are asked questions about rural living, they may revert to restricted code or less elaborate language when talking about this familiar environment or becoming more relaxed towards the end of the interview [29].

While it has been postulated that variations in word choice and use of language can occur related to social class [40], differences in rural and urban educational opportunities may also influence language expression. European studies suggest that students from disadvantaged backgrounds lack access to educational opportunities and role models that adequately prepare them for tertiary education [41]. In Australia, it is often the newly graduated, less experienced teachers who are placed in rural schools and this may have an impact on the quality of the educational opportunities available in some rural and remote locations, regardless of the intelligence of the students. While it is acknowledged that the practice of medicine requires good communication skills, an emphasis on well developed communication skills at selection may preclude rural applicants unnecessarily and overlooks the view that professional communication skills can be taught during the course [42]. An undue focus on language skills could impact on the numbers of rural and remote background medical students, which is a well recognised factor in graduating medical doctors who are likely to practice in rural and remote locations [43].

#### Limitations

There were several limitations to this study including the voluntary nature of recruitment which prevented any theoretical sampling and the limitations posed by a time-limited written exercise. The writing task was compulsory, but its use for research purposes required consent. Perhaps only students who were confident about their academic writing, or particularly interested or motivated, volunteered to submit their essays for analysis. This could have limited our ability to access data from students who may exhibit less developed communication skills. However, the response achieved from all backgrounds is consistent with the demographics of the cohort and analysis across the data set achieved thematic saturation. Therefore, the outcomes of this study provide a valid indication of perceptions, but possibly not the breath of expression among these beginning medical students.

## Conclusion

Despite espousing a commitment to JCU’s rural medicine course, beginning medical students expressed differing perceptions of rural life and practice influenced by their background. Rural background students used every day descriptive language to portray more positive perceptions of practicalities of rural life and medical practice. On the other hand, urban students’ perceptions were more negatively nuanced and notionally constructed, often using key phrases amidst more complex language. These outcomes have several implications for medical schools such as JCU who use interviews as an important part of their selection process. First, admission/selection committees and interviewer pools need to include people from rural and underserved backgrounds. Second, applicants from rural and remote locations may be disadvantaged because of the interviewer’s expectations concerning the use and complexity of language. Additionally, interviewers may be unduly positively influenced by the halo effect induced by an applicant who uses complex language incorporating key phrases. Interviewers need to be trained to recognise these potential points of bias and to listen beyond the language for the detail that indicates an applicants’ suitability for medical courses designed to prepare medical doctors for practicing in underserved locations.

More research is needed to examine the influence of communication in medical student selection, specifically the impact that notional and experiential expression has on interviewers. Additionally, further research following these two cohorts is required to determine how their perceptions of rural medical practice change over their years in medical school and to identify if urban and rural/remote students become more aligned in their views after experiencing significant immersion in rural life and clinical practice.

## References

[1] Hays R, Stokes J, Veitch J (2003). A new socially responsible medical school for regional Australia. Education Health.

[2] Rolfe IE, Pearson SA, O’Connell DL, Dickinson JA (1995). Finding solutions to the rural doctor shortage: the roles of selection versus undergraduate medical education at Newcastle. Aust N Z J Med.

[3] Henry JA, Edwards BJ, Crotty B (2009). Why do medical graduates choose rural careers?. Rural Remote Health.

[4] Walker JH, DeWitt DE, Pallant JF, Cunningham CE. Rural origin plus a rural clinical school placement is a significant predictor of medical students’ intentions to practice rurally: a multi-university study. Rural Rem Health. 2012;12. [online] 22239835

[5] Tolhurst HM, Adams J, Stewart SM: An exploration of when urban background medical students become interested in rural practice. Rural Rem Health. 2006;6. [online] 16544958

[6] Australian Bureau of Statistics (2012). 3235.0 population by age and sex, regions of Australia.

[7] National Rural Health Alliance (2010). Measuring the metropolitan-rural inequity. Fact sheet 23.

[8] Rosenblatt RA, Chen FM, Lishner DM, Doescher MP (2010). The future of family medicine and implications for rural primary care physician supply. Final report 125.

[9] Murray R (2012). Do available predictions of future medical workforce requirements provide a sensible basis for planning?. Med J Aust.

[10] Laven G, Wilkinson D, Beilby J, McElroy H (2005). Empiric validation of the rural Australian medical under-graduate scholarship’ rural background’ criterion. Australian J Rural Health.

[11] Medical Training Review Panel (2010). Thirteenth report.

[12] Australian Institute of Health and Welfare (2014). Medical workforce 2012 National health workforce series no. 8. Cat. no. HWL 54.

[13] Rural Health Workforce Australia (2014). Medical practice in rural and remote Australia: combined rural workforce agencies national minimum data set report as at 30th November 2013.

[14] Laven G, Beilby J, McElroy H, Wilkinson D (2003). Factors associated with rural practice among Australian-trained general practitioners. Med J Aust.

[15] Roseamelia C, Greenwald JL, Bush T, Pratte M, Wilcox J, Morley CP (2014). A qualitative study of medical students in a rural track: views on eventual rural practice. Fam Med.

[16] Barrett FA, Lipsky MS, Nawal Lutfiyya M (2011). The impact of rural training experiences on medical students: a critical review. Acad Med.

[17] Lee Y, Barnard A, Owen C (2011). Initial evaluation of rural programs at the Australian National University: understanding the effects of rural programs on intentions for rural and remote medical practice. Rural Remote Health.

[18] Bore M, Munro D, Powis D (2009). A comprehensive model for the selection of medical students. Med Teach.

[19] Adam J, Bore M, McKendree J, Munro D, Powis D (2012). Can personal qualities of medical students predict in-course examination success and professional behaviour? An exploratory prospective cohort study. BMC Med Educ.

[20] Murray RB, Larkins S, Russell H, Ewen S, Prideaux D (2012). Medical schools as agents of change: socially accountable medical education. Med J Aust.

[21] Laurence CM, Zajac IT, Turnbull DA, Sumner KE, Fleming J (2013). Applicants to the University of Adelaide medical school: influences, motivation and alternative career choices. Focus Health Professional education: A Multidisciplinary J.

[22] Wilson IG, Roberts C, Flynn EM, Griffin B (2012). Only the best: medical student selection in Australia. Med J Aust.

[23] King N, Cassell C, Symon G (2004). Using templates in the thematic analysis of text. Essential guide to qualitative methods in organisational research.

[24] Crabtree BF, Miller WL, Crabtree BF, Miller WL (1999). Using codes and code manuals. Doing qualitative research.

[25] Ray RA (2012). General practice registrars’ intentions to pursue a rural general practice career: a retrospective study. Project report.

[26] Brooks J, King N (2012). Qualitative psychology in the real world: the utility of template analysis. British Psychological Society Annual Conference, 18th - 20th April 2012 London.

[27] Australian Standard Geographical Classification - Remoteness Area (ASGC-RA) http://www.health.gov.au/internet/otd/Publishing.nsf/Content/RA-intro 10.1111/j.1444-0938.2011.00590.x 21426397

[28] Sarantakos S (2000). Quality of family life on the farm. J Family Studies.

[29] Emmitt M, Komesaroff L, Pollock J, Zbaracki (2010). Language and learning.

[30] Jones M, Humphreys J, Prideaux D (2009). Predicting medical students’ intentions to take up rural practice after graduation. Med Educ.

[31] Hegazi I, Wilson I (2013). Medical education and moral segmentation in medical students. Med Educ.

[32] Chen DCR, Kirshenbaum DS, Yan J, Kirshenbaum E, Aseltine RH (2012). Characterizing changes in student empathy throughout medical school. Med Teach.

[33] Coria A, McKelvey TG, Charlton P, Woodworth M, Lahey T (2013). The design of a medical school social justice curriculum. Acad Med.

[34] Lambe P, Bristow D (2011). Predicting medical student performance from attributes at entry: a latent class analysis. Med Educ.

[35] Laurence CO, Turnbull DA, Briggs NE, Robinson JS (2010). Applicant characteristics and their influence on success: results from an analysis of applicants to the University of Adelaide Medical School, 2004–2007. Med J Aust.

[36] Max BA, Gelfand B, Brooks MR, Beckerly R, Segal S (2010). Have personal statements become impersonal? An evaluation of personal statements in anesthesiology residency applications. J Clin Anesth.

[37] Prideaux D, Roberts C, Eva K, Centeno A, Mccrorie P, Mcmanus C (2011). Assessment for selection for the health care professions and specialty training: consensus statement and recommendations from the Ottawa 2010 conference. Med Teach.

[38] Govaerts MJB, Schuwirth LWT, Van der Vleuten CPM, Muijtjens AMM (2011). Work-based assessment: effects of rater expertise. Advances Health Sci Educ.

[39] Plous S (1993). The psychology of judgement and decision making.

[40] Bernstein B (2003). Class, codes and control: applied studies towards a sociology of language.

[41] Chowdry H, Crawford C, Dearden L, Goodman A, Vignoles A (2010). Widening participation in higher education: analysis using linked administrative data. Discussion paper No 4991.

[42] Prowis D (2009). Personality testing in the context of selecting health professionals. Med Teach.

[43] Royston PJ, Mathieson K, Leafman J, Ojan-Sheehan O: Medical student characteristics predictive of intent for rural practice. Rural Rem Health. 2012;12. Available: http://www.rrh.org.au 22873948

